# The Effectiveness of Low-Density Lipoprotein/Fibrinogen Apheresis in Promoting Wound Healing of No-Option Chronic Limb-Threatening Ischemia Foot Ulcers with Wound, Ischemia, and Foot Infection (WIfI) Wound Grade 3: A Single-Center Retrospective Analysis

**DOI:** 10.3390/jcm14082589

**Published:** 2025-04-09

**Authors:** Miki Fujii, Haruna Hirai, Rei Tomyo, Ryo Mizobuchi, Ai Omori, Rica Tanaka, Hiroshi Mizuno

**Affiliations:** 1 Department of Plastic and Reconstructive Surgery, Tokyo Medical University, Tokyo 160-0023, Japan; 2 Department of Plastic and Reconstructive Surgery, Juntendo University School of Medicine, Tokyo 113-8421, Japan; 3 Department of Plastic and Reconstructive Surgery, Juntendo University Nerima Hospital, Tokyo 177-8521, Japan; 4 Division of Regenerative Therapy, Juntendo University Graduate School of Medicine, Tokyo 113-8421, Japan

**Keywords:** chronic limb-threatening ischemia (CLTI), Rheocarna^®^, no-option CLTI, wound healing, foot ulcer

## Abstract

**Background:** Chronic limb-threatening ischemia (CLTI) is a severe condition associated with high mortality and amputation rates, particularly in patients with diabetes, renal failure, or severe vascular disease. In cases where revascularization fails or is not possible, adjunctive therapies can improve the treatment outcomes. Therefore, this single-center retrospective study aimed to evaluate the effectiveness of low-density lipoprotein/fibrinogen apheresis (Rheocarna^®^) in promoting wound healing in patients with no-option CLTI, focusing on large wounds. **Methods:** We examined the data of 32 CLTI ulcers treated with Rheocarna^®^ from 2021 to 2024. **Results:** The outcomes in 25 cases (78.1%) were rated as excellent or good, and the outcomes of 11 (73.3%) wound, ischemia, and foot infection (WIfI) wound-3 ulcers were excellent or good. Overall, 75% of the CLTI ulcers achieved wound healing without major amputation. Predictive factors for successful wound healing included age, baseline skin perfusion pressure, and wound grade (WIfI classification). A skin perfusion pressure threshold of 28.5 mmHg (WIfI ischemic grade 3) was a significant predictor of positive outcomes. **Conclusions:** Our results support the use of Rheocarna^®^ as a viable adjunctive therapy in managing refractory large ischemic ulcers and preventing major amputations.

## 1. Introduction

The number of patients with chronic limb-threatening ischemia (CLTI), an advanced stage of peripheral arterial disease (PAD), is increasing due to an aging population and the global epidemics of diabetes and renal failure. CLTI is a clinical syndrome defined by the presence of PAD in combination with rest pain, gangrene, or lower limb ulceration >2 weeks duration. The annual incidence of CLTI is 50–100 cases per 100,000 individuals, and the mortality rates are approximately 20% at 6 months after onset [1]. CLTI is associated with increased mortality, a higher risk of amputation, and impaired quality of life, imposing a significant economic burden on healthcare systems. The standard treatment for CLTI involves antiplatelet or anticlotting medications and revascularization with distal bypass surgery or endovascular treatment (EVT). However, in some cases, especially in patients with diabetes or those undergoing hemodialysis, traditional revascularization procedures cannot successfully improve the circulation adequately to allow for wound healing, resulting in what is known as “no-option CLTI” [2,3]. In such cases, effective adjunct therapy is necessary to preserve limb function.

**Figure 1:**
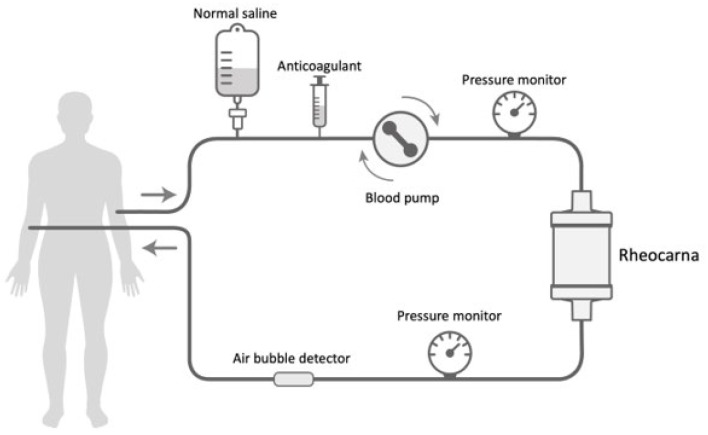
Overview of treatment with Rheocarna^®^.

A new low-density lipoprotein/fibrinogen apheresis (Rheocarna^®^; Kaneka Corporation, Osaka, Japan), which provides blood purification therapy, has been developed as an adjunctive therapy for no-option CLTI. This device selectively removes LDL-cholesterol and fibrinogen, which cause atherosclerosis of the lower-extremity arteries, resulting in reduced plasma viscosity, improved hemorheology, and, finally, improved circulation. Additionally, Rheocarna^®^ can improve microvascular dysfunction by improving endothelial cell dysfunction (Figure 1). Although some studies have demonstrated the safety and effectiveness of Rheocarna^®^ in treating no-option CLTI [4,5,6,7,8,9,10,11], these reports have limitations, such as being single-case studies or the relatively small size of the target ulcers. In clinical practice, most refractory and costly foot ulcers are severely ischemic and involve large amounts of foot tissue. This study aimed to demonstrate the effectiveness of Rheocarna^®^ in treating severe clinical stages of no-option CLTI, focusing on large wounds and assessing the predictive factors for wound healing success with Rheocarna^®^.

## 2. Materials and Methods

### 2.1. Study Design

In this single-center retrospective observation study, we investigated 41 cases (30 men, 11 women) of 45 refractory ischemic foot ulcers treated with Rheocarna^®^ from April 2021 to July 2024 at our university in Tokyo, Japan. The study examined the limbs of patients presenting with foot ulcers caused by CLTI (ischemic tissue loss due to atherosclerotic disease), vasculitis (inflammatory non-atherosclerotic), and cholesterol crystal embolism (CCE).

Data on the following demographic and clinical characteristics of the study participants were collated:

1. Patient background: age, sex, comorbidities [including diabetes mellitus (DM) and collagen disease], and hemodialysis treatment (HD).
2. Wound status before treatment: classified using the Society for Vascular Surgery Wound, Ischemia, and Foot Infection (WIfI) classification system [12]: wound grades 0–3 (W0–3], ischemic grades 0–3 (I0–3), and foot infection grades 0–3 (fI 0–3) and clinical stages (stage 1–5).
3. Blood supply: assessed through evaluating the baseline skin perfusion pressure (SPP) (mm Hg).
4. Revascularization methods: distal bypass surgery, EVT, or no indication of revascularization.
5. Outcome: excellent, good, or poor.

### 2.2. Ethics Approval and Consent to Participate

This study was performed in accordance with the Declaration of Helsinki and was approved by the Ethics Committee of our university. This retrospective study did not include human biological specimens; therefore, the need for written informed consent was waived in accordance with the ethical guidelines for medical and health research involving human participants in Japan.

### 2.3. Diagnosis

All patients were assessed and treated by our multidisciplinary team. Patients with suspected ischemic foot ulcers were examined by plastic surgeons for the presenting wound and by vascular surgeons and cardiologists for the blood supply. Patients with suspected foot ulcers due to vasculitis were also evaluated by a rheumatologist. CLTI was diagnosed when PAD, combined with rest pain, gangrene, or lower limb ulceration >2 weeks duration, was present [13]. PAD was suspected if the pulse was undetectable in either the dorsalis pedis or posterior tibial arteries, either on palpation or on using a Doppler stethoscope, and if the ankle–brachial index was <0.9 and the SPP was <40 mm Hg [14]. The SPP was measured on the dorsal and plantar aspects of the foot at the point between the bases of the first and second toes, and the mean value was used for analysis. The SPP was measured using a PAD4000 (Kaneka Corporation, Osaka, Japan) with a laser Doppler probe, with the patient lying in a supine position at room temperature (24–26 °C). Patients who were diagnosed with PAD were examined using computed tomography–angiography, duplex ultrasonography, and angiography. All ulcers were classified using the WIfI classification system [12]. SPPs ranging from 40 to 49 mmHg and from 30 to 39 mm Hg and ≤30 mm Hg were classified as WIfI I-1, WIfI I-2, and WIfI I-3, respectively [15]. Vasculitis, a non-atherosclerotic chronic vascular condition, was diagnosed using angiography and according to the general status, assessed by the rheumatologist, based on the specified guidelines [16]. Even if patients had a diagnosis of collagen disease, if the vascular surgeon, cardiologist, and/or rheumatologist found that the foot ulcer was caused by atherosclerotic stenosis or occlusion, not vasculitis, the patient was categorized as having CLTI. CCE was diagnosed with skin lesions, such as blue toes and/or livedo reticularis, eosinophilia (eosinophil count 400/μL), imaging delineating aortic atheroma or aneurysm, or other multiorgan disorders, including those of the kidneys. Definitive diagnosis with skin or muscle biopsy was performed if possible.

### 2.4. Treatment

After the diagnosis of CLTI or vasculitis, antiplatelet or anticlotting medications were prescribed. The patients’ general condition, including diabetes control, renal function, and collagen disease, was controlled by each specialist. Plastic surgeons provided wound treatment, including ointments, wound dressings, and specialized offloading footwear. For patients with severe infection (WIfI infection grade 3), emergency surgical debridement was performed either before revascularization or immediately after revascularization. Based on our previous study, debridement was first performed to control the infection if the preoperative C-reactive protein level was >40 mg/L [17]. In other cases, once revascularization had improved the blood supply, the necrotic or infected tissue was debrided. Patients with infected ulcers were treated by preoperative empirical antibiotic therapy, and the prescribed antibiotics were subsequently modified according to the culture results. Cultures were obtained from deep tissues, when possible. In patients with ulcers and suspected osteomyelitis, bacterial cultures were obtained from the bone during debridement. The duration of antibiotic administration was determined based on the Infectious Diseases Society of America Guidelines [18]. The timing and methodology of revascularization were decided based on the wound size, infection status, PAD severity, and patient’s general condition. The severity of aortoiliac disease was assessed using the TransAtlantic Inter-Society Consensus (TASC) II classification [1], and the severity of femoropopliteal, infrapopliteal, and inframalleolar artery lesions was graded using the Global Limb Anatomic Staging System (GLASS) [13]. If sufficient blood supply for wound healing was not achieved even after revascularization, or if there was no indication of revascularization, treatment using Rheocarna^®^ was initiated for these patients. Before starting treatment with Rheocarna^®^, the blood supply was assessed again based on the SPP, and this value was set as the baseline SPP. Blood perfusion treatment with Rheocarna^®^ was performed at a maximum flow rate of 200 mL/min for 1–2 h per session. The treatment was administered twice weekly, avoiding consecutive days. For patients on hemodialysis, treatment was performed before hemodialysis or on a day without hemodialysis. Vascular access was achieved through a shunt in patients on hemodialysis and through an indwelling catheter or venipuncture in patients who were not on hemodialysis. Rheocarna^®^ treatment continued for a maximum of 12 weeks (24 times). If the ulcer healed before 12 weeks, the treatment with Rheocarna^®^ was stopped.

**Figure 2:**
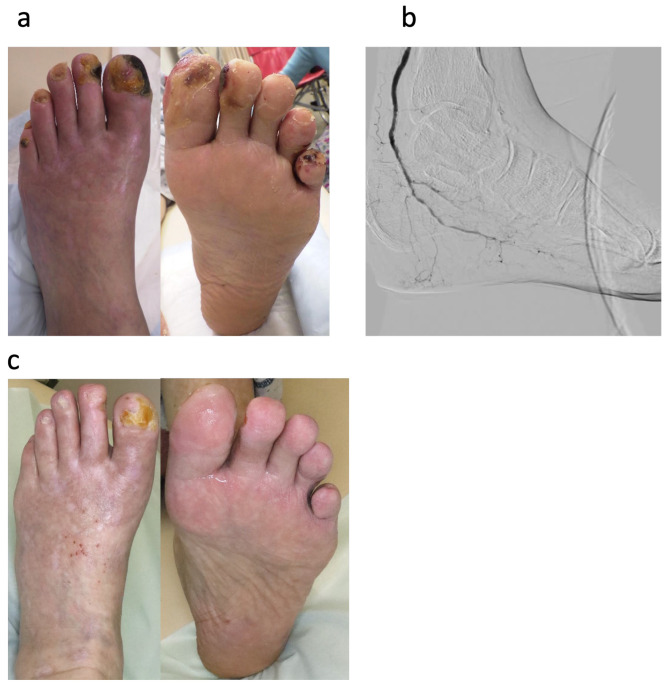
Case 1, 69-year-old male with left CLTI DM, HD excellent case. (**a**) Foot status before Rheocarna^®^ treatment: Although the patient was treated with EVT one month before, his wound did not heal, because he did not have enough circulation. SPP 17 mmHg. WIfI classification: W2, fI0, I3, clinical stage 4; (**b**) Angiography after EVT: blood supply was not sufficient in the forefoot lesion. (**c**) Foot status after 12 weeks of Rheocarna^®^ treatment: After starting Rheocarna^®^ treatment, the foot became warmer, its color improved, and pain decreased. We started maintenance debridement at the outpatient clinic. After 12 weeks of Rheocarna^®^ treatment, the wound showed complete healing.

**Figure 3:**
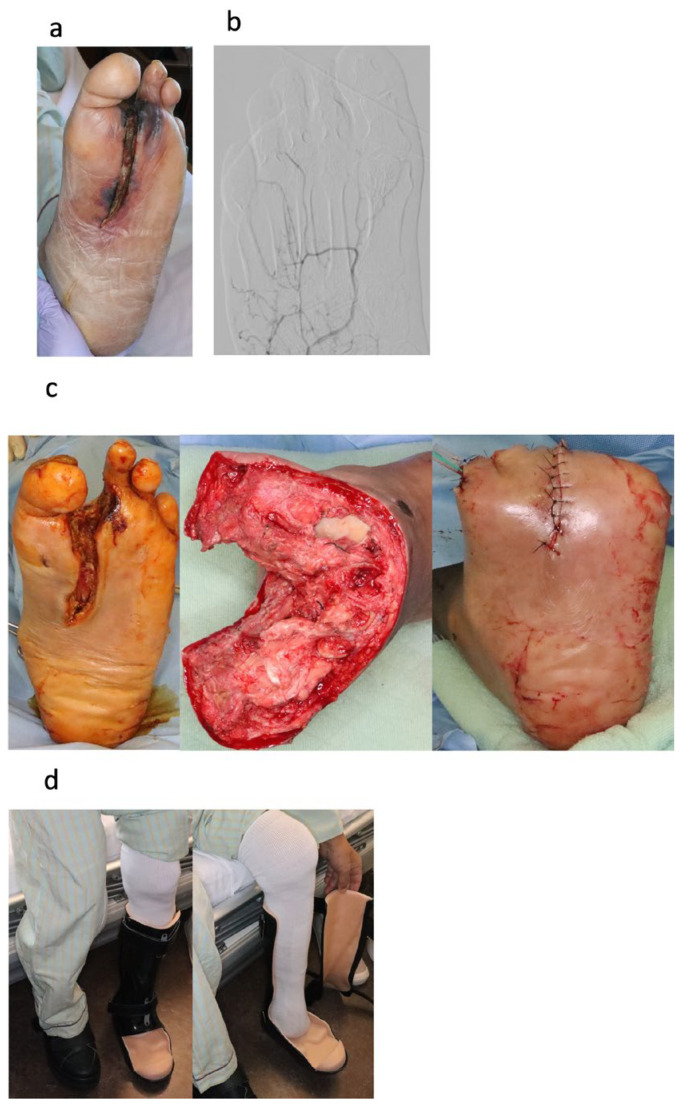
Case 2, 65-year-old male with left CLTI DM, HD excellent case. (**a**) Foot status before Rheocarna^®^ treatment: Although treated with EVT, the patient’s wound did not heal, because he did not have sufficient circulation. The infected 2nd toe was debrided after EVT. SPP 26 mmHg. WIfI classification: W3, fI0, I3, clinical stage 4. (**b**) Angiography after EVT: blood supply was not sufficient in the forefoot lesion. (**c**) Foot status after 6 weeks of Rheocarna^®^ treatment: After starting Rheocarna^®^ treatment, the foot became warmer, its color improved, and pain decreased. We performed wound closure using a flap that was vascularized with a lot of collateral vessels. (**d**) Status 2 months after Rheocarna^®^ treatment: After 2 months, the wound was completely healed. He could walk with customized footwear.

The exclusion criteria for Rheocarna^®^ treatment included malignancy, bleeding tendencies such as intestinal bleeding or intracranial hemorrhage, infected wounds, angiotensin-converting enzyme inhibitor medication, severe anemia, body weight < 40 kg, or hypofibrinogenemia. Before using Rheocarna^®^, fecal occult blood tests were performed to exclude intestinal bleeding, if possible. When the clinical picture confirmed improved circulation, such as through decreased pain, warming foot, improved foot color, or increased SPP, we started maintenance debridement for small wounds of WIfI wound grade 2 during Rheocarna^®^ treatment (Figure 2). For large wounds of WIfI wound grade 2 and 3, we performed surgical wound closure with foot tissue with good circulation (Figure 3). After wound closure, we continued treatment with Rheocarna^®^ until complete wound healing was confirmed.

### 2.5. Outcome Measures

The outcome (excellent, good, poor) was evaluated after 12 weeks of Rheocarna^®^ treatment. If the ulcer had healed before 12 weeks, the outcome was evaluated at that time. If we confirmed wound healing within 12 weeks, we categorized the outcome as excellent. If we confirmed an improvement in wound size or increased blood flow, the outcome was categorized as good. The assessment of blood flow was confirmed by angiography if possible. If we could not perform an angiography, we made a clinical assessment. If there was no improvement in wound size or vascular status, the outcome was classified as poor. Additionally, the wound status was evaluated 4 weeks after Rheocarna^®^ treatment.

### 2.6. Statistical Analysis

Data are presented as medians and quartiles for continuous variables and as incidence and frequency for categorial variables. Two univariate analyses were performed to assess the association between outcomes and valuables. The statistical analysis between continuous variables and the outcomes of all patients and only patients with CLTI was performed with the Kruskal–Wallis test, and those between categorial variables and outcomes were performed with Fisher’s exact test. The factors related to outcomes were evaluated using multivariate analysis for the entire ulcer sample. The analysis was limited to patients with CLTI. The response variable was the outcome (poor vs. good vs. excellent), and the explanatory variables included age, diagnosis, revascularization, sex, presence of diabetes, presence of collagen disease, baseline SPP, presence of dialysis, WIfI classification, and the W (wound) and I (ischemia) components of the WIfI classification. A logistic regression model was used for the evaluation. In this analysis, the Wald test was applied to evaluate the significance of differences in postoperative parameters between the two groups, and the significance level was set to 0.05. All assessments were performed with R version 4.4.0 (R Core Team 2024, Vienna, Austria). To evaluate the prognostic discriminatory ability of the baseline SPP, a receiver operating characteristic (ROC) analysis was performed.

## 3. Results

### 3.1. Patient and Wound Characteristics

Among the 41 cases (30 men, 11 women) of 45 refractory ischemic foot ulcers included in this study, the Rheocarna^®^ treatment was interrupted immediately after starting in 4 patients with CLTI (less than two applications of Rheocarna^®^). The reasons were gastrointestinal bleeding in one, wound infection in one, death in one, and transfer to another hospital in one patient. Because a fecal occult blood test was not performed for the patient with gastrointestinal bleeding before Rheocarna^®^ treatment, we could not exclude the case a priori. Thus, four patients were excluded from this study. Thirty-seven patients with 41 ischemic foot ulcers were finally examined in the study. Table 1 describes the characteristics and wounds of the enrolled patients: 26 patients (70.3%) were men, 27 (73%) had DM, 17 (45.9%) were undergoing dialysis, and 7 (18.9%) had autoimmune disease. All patients had ambulatory ability. Of the 41 foot ulcers, 32 (78%) were diagnosed with CLTI, 5 (12.2%) with CCE, and 4 (9.8%) with vasculitis. All 26 revascularizations were performed using EVT (25 for CLTI, 1 for vasculitis), and no distal bypass surgery was performed. During endovascular therapy (EVT), the primary stenting strategy was generally applied to aortoiliac lesions, whereas the provisional stenting strategy was usually applied to femoropopliteal lesions. The device used in each arterial lesion was selected at the physician’s discretion, determined by the anatomical severity. An atherectomy device was not used, because it was not approved in Japan during the study period. Angioplasty with a noncoated balloon was performed to treat the infrapopliteal and inframalleolar artery lesions. Overall, 15 foot ulcers (36.6%) (7 CLTI, 5 CCE, and 3 vasculitis cases) showed no indication of revascularization. The average baseline SPP in patients with CLTI was 33.15 ± 18 mmHg. The SPP could not be assessed in patients with CCE and vasculitis because of severe pain. Infected ulcers were treated as mentioned above before starting treatment with Rheocarna^®^. The WIfI classifications of the 32 CLTI foot ulcers are shown in Table 2. The following findings were noted: wound grade: W2 = 16 (50%) and W3 = 16 (50%); and ischemic grade: I0 = 6 (18.8%), I1 = 1 (3.1%), I2 = 8 (25%), and I3 = 17 (53.1%). All ulcers had no foot infection [foot infection grade: fI0 = 32 (100%)], and the clinical stage was as follows: stage 1 = 0; stage 2 = 4 (12.5%); stage 3 = 4 (12.5%); and stage 4 = 24 (75%).

### 3.2. Outcome of Rheocarna^®^ Treatment and Wound Status 4 Weeks After Treatment (Table 3)

Of the 41 ulcers, the outcomes in 22 (53.6%) were rated as excellent, 9 (22%) as good, and 10 (24.4%) as poor. Of the 32 CLTI ulcers, the outcomes in 16 (50%) were rated as excellent, 9 (28.1%) as good, and 7 (21.9%) as poor. Focusing on the wound grade of the CLTI cases, the outcomes in 14 (82.4%) of W-2 cases and in 11 (73.3%) of W-3 cases were excellent or good. The outcomes for all CCE ulcers were rated as excellent. For the four vasculitis ulcers, the outcomes were rated as excellent for 1 (25%) and poor for 3 (75%). Regarding wound status at 4 weeks after Rheocarna^®^ treatment, the outcomes were categorized as excellent and good in 31 ulcers, 12 (29.3%) ulcers healed without amputation, 17 (41.5%) ulcers healed with minor amputation, and 2 healed with below-knee amputation. These two ulcers had severe ischemic gangrene beyond the ankle (W3, I3, fI0, stage 4). Because of the Rheocarna^®^ treatment, the wound healed with below-knee amputation, rather than above-knee amputation. Among the ten ulcers with outcomes categorized as poor, only one ulcer healed with minor amputation. Two ulcers resulted in major amputations, and seven did not heal, including one patient’s death. No adverse events directly related to Rheocarna^®^ treatment were reported.

**Table 3:** Outcome of Rheocarna^®^ treatment and wound status at 4 weeks after treatment.

All Patient; n (%)	CLTI (n = 32)	CCE (n = 5)	Vasculitis (n = 4)	Total (n = 41)
Excellent	16 (50)	5 (100)	1 (25)	22 (53.6)
Healed without amputation	7 (21.9)	4 (80)	1 (25)	12 (29.3)
Minor amputation	8 (25)	1 (20)	0 (0)	9 (22)
Blow-knee amputation	1 (3.1)	0 (0)	0 (0)	1 (2.4)
Good	9 (28.1)	0 (0)	0 (0)	9 (22)
Minor amputation	8 (25)	0 (0)	0 (0)	8 (19.5)
Blow-knee amputation	1 (3.1)	0 (0)	0 (0)	1 (2.4)
Poor	7 (21.9)	0 (0)	3 (75)	10 (24.4)
Minor amputation	1 (3.1)	0 (0)	0 (0)	1 (2.4)
Blow-knee amputation	1 (3.1)	0 (0)	0 (0)	1 (2.4)
Above-knee amputation	1 (3.1)	0 (0)	0 (0)	1 (2.4)
No wound healing	4 * (12.5)	0 (0)	3 (75)	7 (17)
CLTI; n (%)	W-2 (n = 17)	W-3 (n = 15)		
Excellent	12 (70.6)	4 (26.7)		
Healed without amputation	6 (35.3)	1 (6.7)		
Minor amputation	5 (29.4)	3 (20)		
Blow-knee amputation	1 (5.9)	0 (0)		
Good	2 (11.8)	7 (46.7)		
Minor amputation	2 (11.8)	6 (40)		
Blow-knee amputation	0 (0)	1 (6.7)		
Poor	3 (17.6)	4 (26.7)		
Minor amputation		0 (0)		
Blow-knee amputation	0 (0)	1 (6.7)		
Above-knee amputation	0 (0)	1 (6.7)		
No wound healing	2 * (11.8)	2 (13.3)		

* 1 patient died before 4 weeks.

### 3.3. Univariate Analysis

A univariate analysis was performed to assess the associations between outcomes and variables. For all patients, a higher age (*p* = 0.0278), male sex (*p* = 0.0246), and collagen disease (*p* = 0.009) were significantly associated with poor outcomes (*p* < 0.05). In patients with CLTI, a higher age (*p* = 0.0349) and the WIfI wound grade (*p* = 0.0299) were significantly associated with poor outcomes (*p* < 0.05).

### 3.4. Multivariate Analysis

The details of the multivariate analysis are shown in Supplementary Table S1. Since the WIfI classification, as well as the W, I, and fI grades as components of the WIfI classification, are highly correlated with each other, it is worthwhile to add either the WIfI classification or the W, I, or fI grades to the explanatory variables. Table 4 shows that W (wound grade) was the only variable that suggested an association, satisfying *p* < 0.05 in Fisher’s exact test. In this analysis, th W grade was only entered as an explanatory variable. The presence of collagen disease was not included as an explanatory variable due to its high correlation with sex. To account for multicollinearity, explanatory variables related to the dependent variable were selected using the backward stepwise method.

The results for the logistic regression model after model selection are shown in Table 5. The remaining explanatory variables were age, baseline SPP, and W. Although no significant associations were observed at a significance level of 0.05, the following tendencies were suggested due to relatively low *p*-values: An increase in age tended to be associated with a poor prognosis (odds ratio = 0.857; *p* = 0.0515). An increase in baseline SPP tended to be associated with a good or excellent prognosis (odds ratio = 1.099; *p* = 0.111). Cases with a W grade of 3 were suggested to have a tendency toward a poor outcome compared to those with a W grade of 2 (odds ratio = 0.088; *p* = 0.0906).

### 3.5. ROC Analysis of Baseline SPP

**Figure 4:**
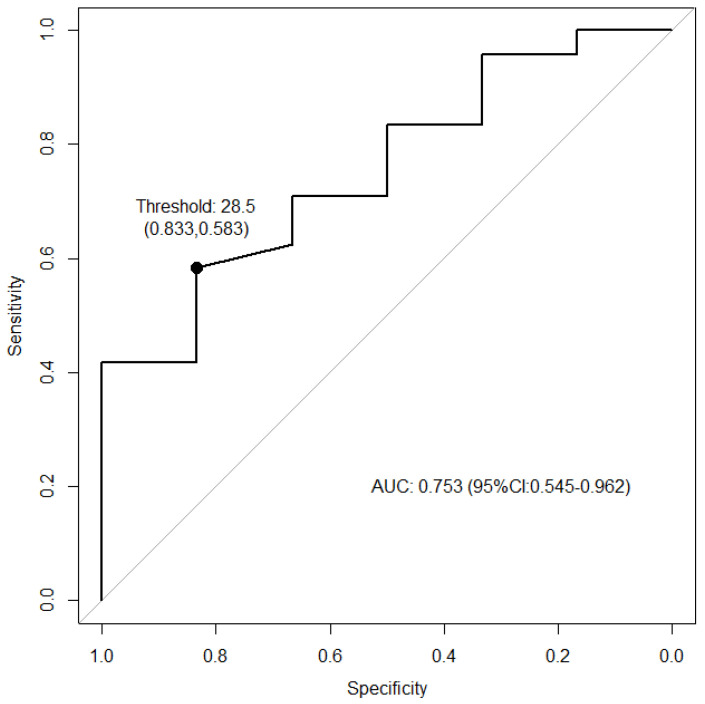
ROC curve (Youden method); threshold, 28.5 mmHg.

The ROC curve of the baseline SPP is shown in Figure 4. The area under the ROC curve, which indicates the discriminatory ability, was 0.753 (95% confidence interval: 0.545–0.962). The cutoff value for the baseline SPP, determined using the Youden method, was estimated to be 28.5 mmHg. When classifying patients with baseline SPPs above the threshold as having a good or excellent prognosis, the sensitivity was 0.583, and when classifying those below the threshold as having a poor prognosis, the specificity was 0.833.

The results before and after the application of the backward stepwise method, as a variable reduction method, are shown. The results after the variable reduction method are the same as in Table 5. For the variable reduction method, explanatory variables were selected to minimize Akaike’s information criteria. For categorical variables among the explanatory variables, the reference level in the multivariate analysis is shown in parentheses.

## 4. Discussion

This study demonstrated the effectiveness of Rheocarna^®^ treatment for severe ischemic and large wounds of no-option CLTI. Predictive factors related to unsuccessful wound healing from Rheocarna^®^ treatment were a higher age, the baseline SPP, and WIfI wound grade 3. The threshold for a poor prognosis of the baseline SPP was 28.5 (sensitivity: 0.583; specificity: 0.833).

EVT for revascularization of the ischemic limb has become increasingly popular and is often the first-line procedure, instead of distal bypass surgery. Advancements in endovascular technology and technique have led to high technical success rates. However, failure may occur due to the absence of distal target vessels, severe calcification, and a heavy plaque burden that results in elastic recoil and early restenosis after EVT. Even after successful revascularization, EVT does not necessarily improve microvascular circulation but can damage the arteries. Advanced disease with occlusion of the pedal arteries, which are commonly used for distal bypasses, can also lead to no indication or failure of distal bypass surgery and culminate in major amputation. Many reports have shown that up to 20% of patients with CLTI are deemed “unreconstructable” with revascularization techniques due to the absence of a viable distal target vessel, viable conduit, or other comorbidities, indicating “no-option CLTI” [2,3].

For the treatment of no-option CLTI, adjunctive therapy has been developed to improve microcirculation. Spinal cord stimulation promotes the activation of cell signaling pathways that cause the release of vasodilatory molecules, leading to a decrease in vascular resistance and relaxation of smooth muscles; this improves the peripheral microcirculatory status [13]. However, spinal cord stimulation involves an invasive procedure. Deep vein arterialization creates a surgical artificial anastomosis between a site of proximal arterial inflow and retrograde venous outflow to deliver tissue perfusion to lower-extremity wounds. This approach has recently been used increasingly to treat no-option CLTI, and its effectiveness has been reported [2,3]. However, this method also necessitates a surgical procedure, posing challenges related to patient suitability and facility availability. Hyperbaric oxygen therapy has been reported to increase the oxygen transport capacity of plasma, improve the function of the leukocyte oxygen-dependent peroxidase system, stimulate progenitor stem cell mobilization and angiogenesis, and lead to increased blood flow and wound healing. However, it also requires special, large equipment and settings [13]. Recently, regenerative therapies, including cell therapy and growth factors, have been developed for therapeutic angiogenesis. Although there is early safety and efficacy trial data, no phase 3 trials have shown this therapy to be effective, preventing its adoption in clinical practice [13].

Rheocarna^®^ is an LDL apheresis adjunct therapy, intended to improve hemorheology [19] while enhancing angiogenesis and vasodilation, thus alleviating inflammation [20]. Traditionally, LDL apheresis has been indicated for familial hypercholesterolemia; however, because of its described mechanism of action, it is also indicated for arteriosclerosis obliterans. Rheocarna^®^ is a direct hemoperfusion-type apheresis device that differs from conventional LDL apheresis therapy and is designed to selectively remove LDL-cholesterol and fibrinogen, which cause atherosclerotic disease of the lower-extremity arteries. This leads to a reduction in plasma viscosity, improves hemorheology, and finally improves circulation. As Rheocarna^®^ is a type of extracorporeal circulation, the procedure can be performed even at small clinics, with no need for a surgical procedure. A multicenter single-arm study with 61 patients with no-option CLTI and foot ulcers (71.5 ± 8 yrs. DM 79%, hemodialysis 80%) demonstrated Rheocarna^®^’s safety and high significance in the treatment of no-option CLTI. The wound healing rate of 45.9% was significantly higher than that of the historical data, and no significant safety concerns were observed [4]. It is worth noting that the average diameter of healed ulcers was within 15 mm, which is relatively smaller than foot ulcers, which become refractory and costly in clinical practice. Kojima reported [10] the clinical benefits of Rheocarna^®^ for 19 patients with no-option CLTI. The wound healing rate was 68.4% and viscosity-related laboratory markers were significantly improved after Rheocarma treatment: low-density lipoprotein–cholesterol (57.0 vs. 43.0 mg/dL; *p* = 0.002), fibrinogen (333 vs. 258 mg/dL; *p* < 0.001), and C-reactive protein (0.99 vs. 0.42 mg/dL; *p* = 0.001). However, 84.2% of the ulcers (n = 16) were Rutherford 5, which corresponds to W2, and only 15.8% (n = 3) were Rutherford 6, which corresponds to W3. Recently, Soga et al. [11] published a multicenter retrospective observational study that analyzed 221 patients (221 limbs) with no-option CLTI. The wound healing rate after 1 year was 60.7%, and the rates of limb salvage, freedom from reintervention, overall survival, and amputation-free survival were 83.4%, 69.2%, 70.2%, and 61.3%, respectively. At baseline, non-ambulatory status, lower ejection fraction, and lower blood albumin levels were independently associated with a lower wound healing rate. However, the majority of ulcers, including those in this study, are small, and only 26.4% are Rutherford 6. This is because their reports were published by cardiologists, not foot surgeons.

In our study, 87.5% of the CLTI ulcers (n = 28/32) were classified as stage 3 or 4, which are associated with a significantly higher incidence of major amputation and delayed wound healing time compared with stages 1 and 2 [21]; 78.1% (n = 25/32) had excellent or good outcomes after 12 weeks treatment with Rheocarna^®^. Four weeks after Rheocarna^®^ treatment, 24 ulcers (75% of CLTI) healed without amputations or with minor amputations. As for the W-3 ulcers, the outcomes in 11 (73.3%) were excellent or good and healed 4 weeks after Rheocarna^®^ treatment. Two ulcers, which led to below-knee amputations, would have required above-knee amputations without Rheocarna^®^ treatment.

All five cases of CCE ulcers had excellent outcomes and healed without or with minor amputations. LDL-A has been commonly used to treat CCE in Japan. A nationwide survey revealed that LDL-A had been used for approximately 13% of patients with CCE after cardiovascular procedures in Japan [22]. Its effectiveness has been reported [23], and our study supports its effectiveness further. However, for vasculitis, only one ulcer healed, whereas the remaining three did not. Ischemic ulcers caused by vasculitis are considerably different from those caused by atherosclerotic CLTI. A recent multicenter retrospective study that compared the clinical outcomes between CLTI and inflammatory non-atherosclerotic ulcers after EVT showed that the inflammatory non-atherosclerotic group had a lower incidence rate of wound healing (0.64-fold, *p* < 0.001) and a higher incidence rate of major EVT reintervention (2.30-fold, *p* = 0.006), and the risk ratio of all-cause mortality was increased by 0.83-fold (*p* = 0.067). Given its minimally invasive nature, Rheocarna^®^ therapy remains a viable option for vasculitis treatment.

Four patients dropped out immediately after starting Rheocarna^®^ treatment in our study. Generally, patients with CLTI are at a continuous risk of a major cardiovascular event, gastrointestinal bleeding, sudden death, wound infection, and major amputation. When an individual first receives a diagnosis of CLTI, the mortality risk is 20–25% over 1 year and increases to approximately 60% over 5 years [24]. Considering these circumstances, our experience is within the forecast. In this context, the observed dropout rate in our study aligns with the expected outcomes for this high-risk population.

SPP measurement is an objective, non-invasive method of diagnosing critical limb ischemia and predicting wound healing, even in patients with severe vascular calcification. In our previous study, we reported that the post-revascularization SPP was a significant predictive factor for wound healing in patients with CLTI [25]. The sensitivity of SPP < 30 mmHg as a diagnostic test of CLTI was 85%, and the specificity was 73%. The overall diagnostic accuracy of SPP < 30 mmHg as a diagnostic test of critical limb ischemia was 79.3% (*p* < 0.002, Fischer’s exact test) [14]. In this study, the ROC analysis suggested that the baseline SPP may be useful for prognosis prediction of Rheocarna^®^ treatment. The estimated cutoff for the baseline SPP was 28.5 mmHg, corresponding to WIfI ischemic grade 3. These findings suggest that ulcers with a baseline SPP of 28.5 mmHg (I-3), whether following revascularization or in cases where revascularization is not indicated, have the potential for wound healing with the introduction of Rheocarna^®^ therapy.

The current use of Rheocarna^®^ is limited to Japan, but this should be accepted into standard clinical treatment for no-option CLTI worldwide. Rheocarna^®^ improves microvascular dysfunction by improving endothelial cell dysfunction. Rheocarna^®^ has the potential for clinical applications for general ischemic diseases caused by arteriosclerosis, such as cardiovascular events or cerebral infarctions.

This study has some limitations. First, the sample size was relatively small, with only 41 cases. However, despite the low number of patients, we successfully demonstrated the effectiveness of Rheocarna^®^ for large, severe ischemic ulcers and suggested factors related to good prognosis. Second, instead of the ankle–brachial pressure, which is the gold standard for assessing vascular status, we used the SPP to evaluate the vascular supply. All the patients with CLTI in this study were Japanese, who have a higher prevalence of renal failure and vascular calcification than other ethnic groups. The ankle–brachial pressure cannot be measured accurately in patients with vascular calcification, such as those with diabetes and end-stage renal disease [26]. Third, we did not measure the outcome as the wound healing rate but showed the wound status after 4 weeks of Rheocarna^®^ treatment. Although small ischemic wounds will heal without surgery if the blood supply improves, large wounds need some kind of surgery, including amputation. Without Rheocarna^®^ treatment, we could not close the wound with amputation, because the tissue was ischemic. Therefore, we intentionally used the term “wound status” instead of “wound healing rate”.

## 5. Conclusions

This study demonstrated that Rheocarna^®^ treatment improved the wound healing of large ulcers at severe clinical stages. These insights can help guide treatment decisions for similar patients in clinical settings. Rheocarna^®^ emerges as a promising, non-invasive treatment for patients who fail standard revascularization procedures, offering a better alternative to other adjunctive therapies.

## Figures and Tables

**Table 1:** Patient and wound characteristics.

	CLTI	CCE	Vasculitis	Total
n (%)				
Patient	28 (75.7)	5 (13.5)	4 (10.8)	37
Sex, male	21 (75)	5 (100)	0 (0)	26 (70.3)
Diabetes	24 (85.7)	3 (60)	1 (25)	27 (73)
Dialysis	17 (60.7)	0 (0)	0 (0)	17 (45.9)
Autoimmune disease	3 (10.7)	0 (0)	4 (100)	7 (18.9)
Foot ulcer	32 (78)	5 (12.2)	4 (9.8)	41
Revascularization method				
No indication	7 (21.9)	5 (100)	3 (75)	15 (36.6)
EVT	25 (78.1)	0 (0)	1 (25)	26 (63.4)
Distal bypass surgery	0 (0)	0 (0)	0 (0)	0 (0)
baseline SPP (mmHg)	33.15 ± 18	no data	no data	

**Table 2:** WIfI classification of CLTI (n = 32).

		n	(%)
Wound grade	0	0	0
	1	0	0
	2	16	50
	3	16	50
Ischemic grade	0	6	18.8
	1	1	3.1
	2	8	25
	3	17	53.1
Foot infection grade	0	32	100
	1	0	0
	2	0	0
	3	0	0
Clinical stage	1	0	0
	2	4	12.5
	3	4	12.5
	4	24	75

**Table 4:** Univariate analysis assessing the association between outcomes and variables.

				Outcome N (%)			
	Categorial Variable		N	Poor	Good	Excellent	Fisher’s Exact Test
All	Diagnosis	CCE	5	0 (0.0%)	0 (0.0%)	5 (100.0%)	0.1428
		CLTI	32	7 (21.9%)	9 (28.1%)	16 (50.0%)	0.2147
		Vasculitis	4	3 (75.0%)	0 (0.0%)	1 (25.0%)	0.0826
	Revascularization	EVT	26	5 (19.2%)	7 (26.9%)	14 (53.8%)	0.5685
		no indication	15	5 (33.3%)	2 (13.3%)	8 (53.3%)	0
	Sex	Female	11	6 (54.5%)	1 (9.1%)	4 (36.4%)	0.0246
		Male	26	3 (11.5%)	6 (23.1%)	17 (65.4%)	0
	DM	No	10	4 (40.0%)	0 (0.0%)	6 (60.0%)	0.1627
		Yes	27	5 (18.5%)	7 (25.9%)	15 (55.6%)	0
	Collagen disease	No	30	4 (13.3%)	7 (23.3%)	19 (63.3%)	0.009
		Yes	7	5 (71.4%)	0 (0.0%)	2 (28.6%)	0
	HD	No	20	6 (30.0%)	4 (20.0%)	10 (50.0%)	0.7392
		Yes	17	3 (17.6%)	3 (17.6%)	11 (64.7%)	0
CLTI	Revascularization	EVT	25	4 (16.0%)	7 (28.0%)	14 (56.0%)	0.2487
		no indication	7	3 (42.9%)	2 (28.6%)	2 (28.6%)	0
	Sex	F	7	3 (42.9%)	1 (14.3%)	3 (42.9%)	0.4254
		M	21	3 (14.3%)	6 (28.6%)	12 (57.1%)	0
	DM	No	5	2 (40.0%)	0 (0.0%)	3 (60.0%)	0.281
		Yes	23	4 (17.4%)	7 (30.4%)	12 (52.2%)	0
	Collagen disease	No	25	4 (16.0%)	7 (28.0%)	14 (56.0%)	0.156
		Yes	3	2 (66.7%)	0 (0.0%)	1 (33.3%)	0
	HD	No	11	3 (27.3%)	4 (36.4%)	4 (36.4%)	0.4022
		Yes	17	3 (17.6%)	3 (17.6%)	11 (64.7%)	
	W grade	0	0	0 (%)	0 (%)	0 (%)	NA
		1	0	0 (%)	0 (%)	0 (%)	NA
		2	17	3 (17.6%)	2 (11.8%)	12 (70.6%)	0.0299
		3	15	4 (26.7%)	7 (46.7%)	4 (26.7%)	0.0299
	I grade	0	6	0 (0.0%)	1 (16.7%)	5 (83.3%)	0.2988
		1	1	0 (0.0%)	0 (0.0%)	1 (100.0%)	1.0000
		2	8	1 (12.5%)	5 (62.5%)	2 (25.0%)	0.0644
		3	17	6 (35.3%)	3 (17.6%)	8 (47.1%)	0.1220
	fI grade	0	32	7 (21.9%)	9 (28.1%)	16 (50.0%)	NA
		1	0	0 (%)	0 (%)	0 (%)	NA
		2	0	0 (%)	0 (%)	0 (%)	NA
		3	0	0 (%)	0 (%)	0 (%)	NA
	Stage	1	0	0 (%)	0 (%)	0 (%)	NA
		2	4	0 (0.0%)	0 (0.0%)	4 (100.0%)	0.1542
		3	4	0 (0.0%)	1 (25.0%)	3 (75.0%)	0.7898
		4	24	7 (29.2%)	8 (33.3%)	9 (37.5%)	0.0543
		5	0	0 (%)	0 (%)	0 (%)	NA
			**Outcome**				**Test**
	**Continuous Valuable**		**Poor**	**Good**		**Excellent**	**Kruskal-Wallis**
All	Age	N	10	9		22	0.0278
		Mean (SD)	76.5 (8.59)	62.1 (11.53)		71.0 (9.92)	
		Min, Max	64, 91	52, 87		52, 86	
		Median (25%, 75%)	74.5 (70.2, 83.0)	58.0 (54.0, 64.0)		72.5 (66.0, 78.5)	
	Baseline SPP	N	6	9		15	0.1208
		Mean (SD)	21.8 (4.00)	30.6 (13.32)		39.2 (7.00)	
		Min, Max	10, 36	13, 59		17, 86	
		Median (25%, 75%)	21.5 (16.0, 26.2)	31.0 (23.0, 33.0)		38.0 (23.0, 52.0)	
CLTI	Age	N	7	9		16	0.0349
		Mean (SD)	77.9 (8.57)	62.1 (11.53)		68.7 (10.47)	
		Min, Max	69, 91	52, 87		52, 86	
		Median (25%, 75%)	76.0 (70.5, 84.0)	58.0 (54.0, 64.0)		70.0 (58.8, 76.2)	
	Baseline SPP	N	6	9		15	0.1208
		Mean (SD)	21.8 (1.00)	30.6 (13.32)		39.2 (1.00)	
		Min, Max	10, 36	13, 59		17, 86	
		Median (25%, 75%)	21.5 (16.0, 26.2)	31.0 (23.0, 33.0)		38.0 (23.0, 52.0)	

For all patients, a higher age, male sex, and collagen disease were significantly associated with poor outcomes (*p* < 0.05). In patients with CLTI, a higher age and the WIfI wound grade were significantly associated with poor outcomes (*p* < 0.05).

**Table 5:** Results for logistic regression model after model selection by backward stepwise method.

Variable	Odds Ratio (95% CI)	*p*
Age	0.857 (0.734–1.001)	0.0515
baseline SPP	1.099 (0979–1.234)	0.1111
W is 3 against 2	0.088 (0.005–1.468)	0.0906

## Data Availability

All data analyzed in this study are available from the corresponding author upon request.

## Supplementary Materials

The following supporting information can be downloaded at: https://www.mdpi.com/article/10.3390/jcm14082589/s1, Table S1: Details of the multivariate analysis.
