# Supplementary material for: The Effectiveness of Low-Density Lipoprotein/Fibrinogen Apheresis in Promoting Wound Healing of No-Option Chronic Limb-Threatening Ischemia Foot Ulcers with Wound, Ischemia, and Foot Infection (WIfI) Wound Grade 3: A Single-Center Retrospective Analysis
Source: J Clin Med. 2025 Apr 9;14(8):2589. doi: 10.3390/jcm14082589 (PMC12027775; doi:10.3390/jcm14082589)
Supplement: Supplementary file 1 [file jcm-14-02589-s001.zip › jcm-3539817-supplementary.pdf]

Supplementary Table S1

Before stepwise Variables		OR (95%CI)	P	After stepwise Variables		OR (95%CI)	P
Age		0.833 (0.681,1.018)	0.0745	Age		0.857 (0.734,1.001)	0.0515
Revascularization	no indication	0.366 (0.006,23.403)	0.6354				
Sex (F)	M	0.780 (0.020,30.744)	0.8944				
DM (No)	Yes	0.679 (0.014,33.976)	0.8461				
HD (No)	Yes	0.353 (0.008,14.737)	0.5845				
Baseline SPP		1.123 (0.973,1.295)	0.1139	baseline SPP		1.099 (0.979,1.234)	0.1111
W (2)	3	0.137 (0.007,2.691)	0.1909	W (2)	3	0.088 (0.005,1.468)	0.0906
